# Gestational diabetes mellitus in previous pregnancy associated with the risk of large for gestational age and macrosomia in the second pregnancy

**DOI:** 10.3389/fendo.2025.1474694

**Published:** 2025-02-03

**Authors:** Ying Wang, Juan Yang, Yuzhen Liu, Ao Yang, Yuqing Deng, Chang Xu, Shilin Zhong

**Affiliations:** ^1^ Center of Obstetrics and Gynecology, Peking University Shenzhen Hospital, Shenzhen, Guangdong, China; ^2^ Institute of Obstetrics and Gynecology, Shenzhen Peking University-Hong Kong University of Science and Technology (PKU-HKUST) Medical Center, Shenzhen, Guangdong, China; ^3^ Peking University Shenzhen Hospital, Shenzhen Key Laboratory on Technology for Early Diagnosis of Major Gynecologic Diseases, Shenzhen, Guangdong, China; ^4^ Intelligent Hospital Research Academy, Peking University Shenzhen Hospital, Shenzhen, Guangdong, China

**Keywords:** large for gestational age, macrosomia, gestational diabetes mellitus, body mass index, gestational weight gain, multipara

## Abstract

**Background:**

Since the implementation of China’s new birth policy, the incidence of large for gestational age (LGA) and macrosomia associated with gestational diabetes mellitus (GDM) has increased. It remains unclear whether a history of GDM in a previous pregnancy raises the risk of LGA or macrosomia in Chinese women planning two or more pregnancies.

**Aim:**

To analyze the association between previous GDM and the risk of LGA and macrosomia in second pregnancy.

**Method:**

A retrospective study was conducted on a cohort of 3,131 women who had experienced two consecutive singleton births. The incidences of LGA and macrosomia in the second pregnancy were compared between women with and without previous GDM. The relationship between previous GDM and the occurrence of LGA and macrosomia was analyzed using multivariate logistic regression and stratified analysis.

**Results:**

The incidence of LGA and macrosomia during the second pregnancy was significantly higher in women with previous GDM (22.67% and 10.25%, respectively) compared to those without prior GDM (15.34% and 5.06%, respectively) (P < 0.05). After adjusting for potential confounders, previous GDM was significantly associated with LGA (aOR: 1.511, 95% CI: 1.066-2.143) and macrosomia (aOR: 1.854, 95% CI: 1.118-3.076) in the second pregnancy. Stratified analysis revealed that these associations were present only in women without previous LGA, those with GDM, appropriate gestational weight gain (AGWG), non-advanced maternal age, and male newborns during the second pregnancy (*P* < 0.05). Compared to excessive GWG (EGWG), AGWG correlated with lower risks for LGA and macrosomia during the second pregnancy in women without prior GDM, an association not observed in those with previous GDM. Among women without previous GDM, if the pre-pregnancy BMI is normal, the risk of LGA and macrosomia is significant lower in AGWG compared with EGWG (*P*< 0.001), while this difference was no significant among women with prior GDM (*P*>0.05).

**Conclusion:**

Previous GDM is strongly linked to LGA and macrosomia in subsequent pregnancies. However, this relationship is influenced by GWG, prior LGA history, fetal sex, and maternal age. Managing weight alone may not sufficiently reduce the risk of LGA or macrosomia for women with a history of GDM.

## 1 Introduction

Large for gestational age (LGA) refers to infants whose birth weight exceeds the 90th percentile for their gestational age and sex, while macrosomia is defined as a birth weight of 4000g or more. In China, the incidence of LGA ranges from 7.4% to 16.8% (1, 2), and macrosomia affects 4.0% to 9.2% of infants (1, 3). Both LGA and macrosomia are associated with elevated risks of emergency cesarean sections, prolonged second stages of labor, shoulder dystocia, birth canal lacerations, and neonatal birth injuries (4, 5). Additionally, they pose potential long-term risks of obesity (6) and diabetes (7). Reducing the incidence of LGA and macrosomia is thus essential for maternal and child health. Known risk factors include gestational diabetes mellitus (GDM) (8), inter-pregnancy weight changes (9, 10), prolonged pregnancy intervals (11), pre-pregnancy overweight or obesity (12, 13), excessive weight gain during pregnancy (12, 14), advanced maternal age (1), multiparity (15), and fetal sex (1).

GDM is a kind of diabetes diagnosed in pregnancy, and its prevalence in China is as high as 14.8% to 16.8% (2, 16). With the increase of multipara and/or advanced pregnancies in China, the risk of GDM also rises. The association between GDM in a previous pregnancy and the risk of LGA in a second pregnancy has been suggested by a 2014 study in the United States (17). However, this particular study did not investigate the risk of macrosomia. Conversely, a recent Chinese study found no significant association between prior GDM and the risk of macrosomia in a second pregnancy, and it also did not examine the LGA risk (18). In September 2020, the growth standard curves of birth weight of Chinese newborns of different gestation was published (19), allowing for more accurate diagnosis of LGA. Thus, it is crucial to investigate the risk factors for LGA and macrosomia using these updated criteria in the Chinese population. A retrospective analysis of clinical data from our center aims to explore the relationship between GDM in a previous pregnancy and the risk of LGA and macrosomia in a subsequent pregnancy.

## 2 Materials and methods

### 2.1 Study design and population

This retrospective study comprised pregnant women who delivered two consecutive singletons at Peking University Shenzhen Hospital from January 2002 to March 2024. The inclusion criteria were: both pregnancies reached 28 weeks of gestation or later, involved singleton pregnancies, and maternal age between 18 and 50 years. The exclusion criteria included: stillbirth, fetal malformation in either pregnancy, multiple pregnancies, pregestational diabetes mellitus, and other pregnancy complications such as chronic hypertension, preeclampsia, intrahepatic cholestasis, or severe cardiac or renal disease in the second pregnancy. Cases lacking information on GDM diagnosis, pre-pregnancy body mass index (BMI), weight gain during pregnancy, and newborn birth weight were also excluded. Eligible cases that met both inclusion and exclusion criteria were selected from the hospital’s medical records. Participants with two deliveries were matched by name, ID number, and delivery time. Data such as age, height, pre-pregnancy BMI, gestational weight gain, nationality, parity, delivery method, gestational age at delivery, neonatal birth weight, neonatal gender, and GDM status were collected from both the hospital’s medical record system and the Shenzhen Maternal and Child Health Care System. This study received approval from the Ethics Committee of Peking University Shenzhen Hospital (No. 2023-103-1).

### 2.2 Diagnostic criteria and definitions of index

According to IADPSG criteria (20), GDM is diagnosed via a 75g oral glucose tolerance test if any of the following plasma glucose values are met: a fasting plasma glucose level of ≥5.1 mmol/L, or 1-h and 2-h plasma glucose levels of ≥10.0 mmol/L and ≥8.5 mmol/L, respectively. LGA was defined as a newborn whose birth weight exceeds the 90th percentile for their corresponding gestational age and sex, according to the Growth standard curves of birth weight of Chinese newborns of different gestation (19). Macrosomia is diagnosed if a newborn’s birth weight is equal to or greater than 4000g.

Body Mass Index (BMI) is calculated by dividing weight (kg) by height squared (m²). According to the standard of Chinese population (21), a BMI of less than 18.5 kg/m² is classified as underweight, a BMI between 18.5 kg/m² and 24 kg/m² as normal weight, a BMI between 24 kg/m² and 28 kg/m² as overweight, and a BMI over 28 kg/m² as obese. The inter-pregnancy change of BMI (IPCB) is determined by subtracting the pre-pregnancy BMI of the previous pregnancy from the pre-pregnancy BMI of the subsequent pregnancy. The inter-pregnancy interval (IPI) is the period (in months) between the end of one pregnancy and the start of the next. Gestational weight gain (GWG) is calculated by subtracting pre-pregnancy weight from the weight before delivery. According to the Standard of Recommendation for Weight Gain During Pregnancy (WST801-2022) (22), appropriate GWG (AGWG) is: 11.0 to 16.0 kg for individuals with a pre-pregnancy BMI of less than 18.5 kg/m², 8.0 to 14.0 kg for a pre-pregnancy BMI of under 24 kg/m², 7.0 to 11.0 kg for a pre-pregnancy BMI of under 28 kg/m², and 5.0 to 9.0 kg for those with a pre-pregnancy BMI over 28 kg/m². GWG below these ranges is classified as insufficient (IGWG), while values above are deemed excessive (EGWG).

### 2.3 Statistical analysis

Data were analyzed using SPSS 26.0 statistical software. Categorical variables were presented as [n (%)], and assessed with the chi-squared test. Continuous data were expressed as mean ± SD, and normality was evaluated using the Shapiro-Wilk test. Normally distributed variables were compared using the student’s *t*-test, while non-normally distributed variables were reported as median (interquartile range; IQR) and compared using the Mann-Whitney *U* test. Multivariable logistic regression models were employed to explore the association between previous GDM and the incidence of LGA and macrosomia in subsequent pregnancies. Stratified logistic multivariate analysis was conducted to examine the impact of previous GDM on LGA and macrosomia in the second pregnancy across groups divided by previous LGA, GDM, maternal age, sex of the newborn, and gestational weight gain (GWG) in the second pregnancy. A P-value of less than 0.05 was considered statistically significant.

## 3 Results

### 3.1 The characteristics of study population

**Figure 1:**
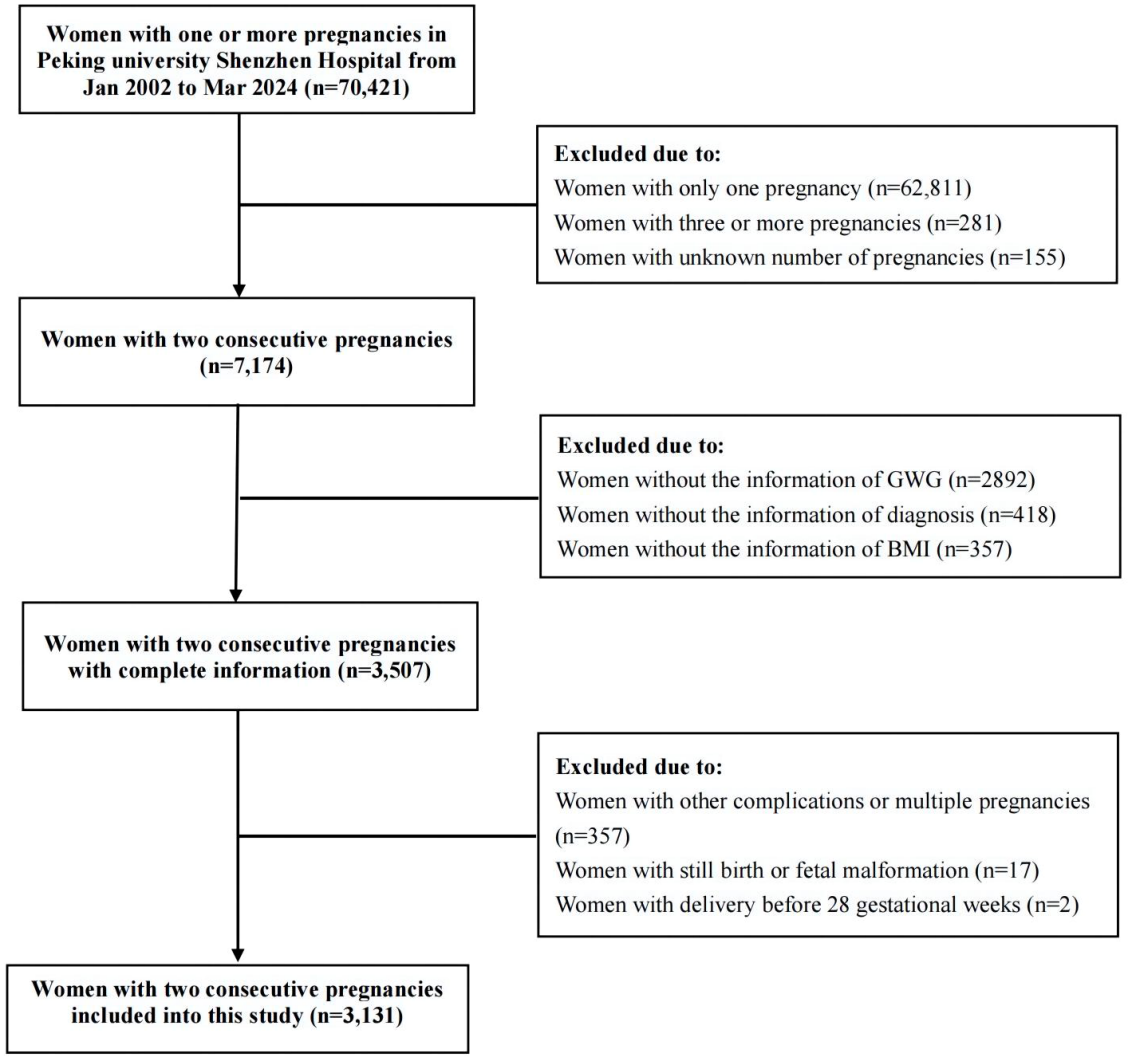
Flow chart showing inclusion and exclusion in this study. GWG: gestational weight gain; BMI: body mass index.

This study included a total of 3,131 pregnant women (**Figure 1**). In their previous pregnancies, 322 cases (10.28%) had GDM, 313 cases (10.00%) had LGA, and 135 cases (4.31%) had macrosomia. During their second pregnancies, 501 cases (16.00%) had GDM, 504 cases (16.10%) had LGA, and 175 cases (5.59%) had macrosomia. The average birth weight in the second pregnancy (3304.66 ± 423.57g) was significantly higher than in the previous pregnancy (3237.96±439.22g) (*t*=6.117, *P*<0.001). Additionally, the incidence of LGA was significantly higher in the second pregnancy compared to the previous one (*χ*2 = 51.352, *P* < 0.001), as was the incidence of macrosomia (*χ*2 = 5.430, *P* = 0.020). In women who experienced GDM during their first pregnancy, the likelihood of developing GDM in their second pregnancy was markedly higher compared to those who did not have GDM initially (P<0.001). No significant differences were observed in the risk of other complications and comorbidities between the groups (P>0.05) (**Supplementary Table S1**).

Given that GWG during the second pregnancy is a crucial confounding factor, we analyzed its association with other risk factors, including prior GDM. The GWG of the second pregnancy in women with a history of GDM (12.08 ± 4.35 kg) was significantly lower than that of women without previous GDM (13.38 ± 4.23 kg) (*P* < 0.001) (**Supplementary Table S2**). Similarly, women with GDM in the second pregnancy had a lower GWG (11.92 ± 4.19 kg) compared to those without GDM in the second pregnancy (13.50 ± 4.23 kg) (*P* < 0.001). However, there was no significant difference in GWG during the second pregnancy between groups categorized by previous LGA, advanced pregnancy, or male newborns in the second pregnancy (*P* > 0.05) (**Supplementary Table S2**).

The median inter-pregnancy change in BMI (IPCB) was 0.80 kg/m² (ranging from -0.04 kg/m² to 1.90 kg/m²). A total of 1319 cases (42.13%) had a stable IPCB (-1.0 kg/m² to 1.0 kg/m²), 375 cases (11.98%) had an IPCB between 2.0 kg/m² and 3.0 kg/m², and 311 cases (9.93%) had an IPCB greater than 3.0 kg/m². The pre-pregnancy BMI of the subjects with GDM in the second pregnancy was 22.34 ± 3.11 kg/m², significantly higher than that of subjects without GDM in the second pregnancy (21.19 ± 2.76 kg/m²) (t=7.708, *P*<0.001).

### 3.2 The risk of LGA and macrosomia in the second pregnancy associated with prior GDM

**Figure 2:**
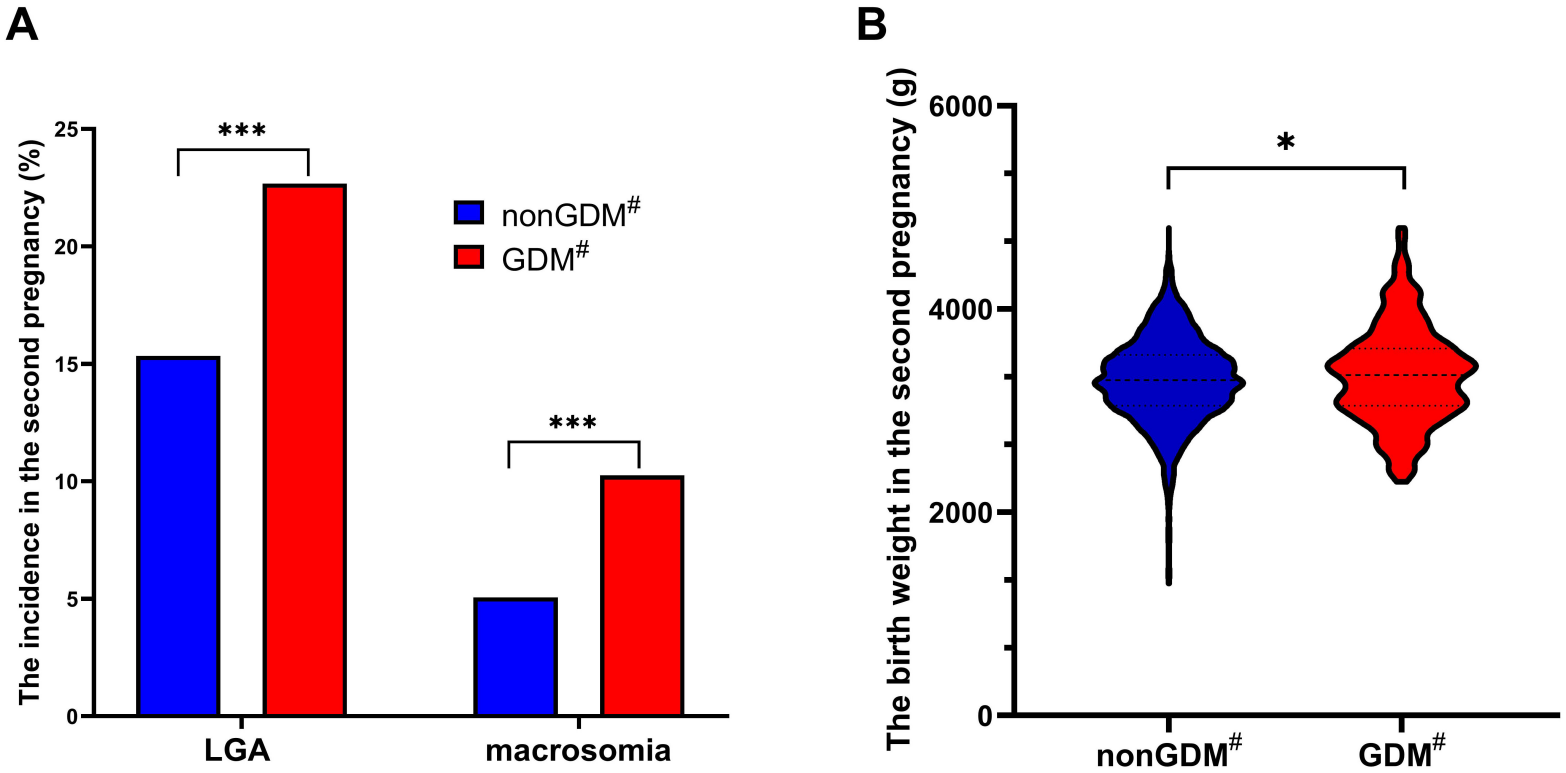
Comparison of the incidence of LGA and macrosomia and the birth weight in the second pregnancy in different groups. The incidence of LGA and macrosomia significantly increased in women with previous GDM compared with those without previous GDM **(A)**; The birth weight of second pregnancy in women with previous GDM was significantly higher than that in women without previous GDM **(B)**; GDM, gestational diabetes mellitus; LGA, large for gestational age; ^*^ *P*<0.05; ^***^ *P*<0.001; ^#^in previous pregnancy.

The incidence of LGA in the second pregnancy for women with prior GDM (22.67%, 73/322) was significantly higher than that in women without previous GDM (15.34%, 431/2809) (*χ*²=11.484, *P* = 0.001) (**Figure 2A**). Similarly, the incidence of macrosomia in the second pregnancy for women with prior GDM (10.25%, 33/322) was significantly higher compared to women without previous GDM (5.06%, 142/2809) (*χ*²=14.765, *P*<0.001) (**Figure 2A**). Additionally, the birth weight of babies born to women with prior GDM (3350.09 ± 474.39g) was significantly higher than those born to women without previous GDM (3299.45 ± 417.13g) (*t*=2.033, *P*=0.042) (**Figure 2B**).

### 3.3 Previous GDM independently contributed to the risk of LGA and macrosomia in the second pregnancy

In the unadjusted analysis, previous GDM, prior LGA, interpregnancy interval (IPI), maternal age, pre-pregnancy BMI, male newborn, GDM, and gestational weight gain (GWG) in the second pregnancy were all significantly associated with LGA in the second pregnancy (*P*<0.05) (**Table 1**), while nationality and IPCB were not significantly associated with LGA (**Supplementary Table S3**). Furthermore, previous GDM and prior LGA, IPCB, GDM, pre-pregnancy BMI, male newborn, and GWG in the second pregnancy were significantly linked to macrosomia in the second pregnancy (*P*<0.05) (**Table 2**), while nationality, IPI and maternal age were not significantly associated with macrosomia (**Supplementary Table S4**).

**Table 1:** Impact of previous GDM and other risk factors on LGA in subsequent pregnancy.

Risk factors	Non-adjusted			Adjusted*		
	*OR*	*95% CI* for *OR*	*P*	*OR*	*95% CI* for *OR*	*P*
GDM in previous pregnancy	**1.618**	**1.222-2.141**	**0.001**	**1.511**	**1.066-2.143**	**0.021**
Male newborn in the second pregnancy	**1.273**	**1.049-1.544**	**0.014**	**1.282**	**1.035-1.589**	**0.023**
LGA in previous pregnancy	**7.167**	**5.590-9.188**	**<0.001**	**6.318**	**4.818-8.285**	**<0.001**
Pre-pregnancy BMI in the second pregnancy	**1.150**	**1.114-1.187**	**<0.001**	**1.130**	**1.084-1.178**	**<0.001**
GWG in the second pregnancy	**1.067**	**1.044-1.091**	**<0.001**	**1.091**	**1.064-1.119**	**<0.001**
IPI	**1.004**	**1.001-1.007**	**0.011**	1.002	0.997-1.006	0.478
Maternal age in the second pregnancy	**1.042**	**1.016-1.070**	**0.002**	1.020	0.985-1.055	0.266
GDM in the second pregnancy	**1.374**	**1.077-1.753**	**0.010**	1.029	0.759-1.395	0.853

GDM, gestational diabetes mellitus; LGA, large for gestational age; IPI, inter-pregnancy interval; GWG, gestational weight gain; *adjusted factors: previous GDM, nationality, previous LGA, IPI, inter-pregnancy change of body mass index, maternal age in the second pregnancy, GDM in the second pregnancy, pre-pregnancy BMI in the second pregnancy, male newborn in the second pregnancy, GWG in the second pregnancy. Numbers with statistical significance were marked in bold.

**Table 2:** Impact of previous GDM and other risk factors on macrosomia in subsequent pregnancy.

Risk factors	Non-adjusted			Adjusted*		
	*OR*	*95% CI* for *OR*	*P*	*OR*	*95% CI* for *OR*	*P*
GDM in previous pregnancy	**2.145**	**1.441-3.192**	**<0.001**	**1.854**	**1.118-3.076**	**0.017**
LGA in previous pregnancy	**7.235**	**5.200-10.066**	**<0.001**	**5.616**	**3.857-8.177**	**<0.001**
Pre-pregnancy BMI in the second pregnancy	**1.186**	**1.134-1.241**	**<0.001**	**1.163**	**1.095-1.234**	**<0.001**
Male newborn in the second pregnancy	**2.510**	**1.779-3.541**	**<0.001**	**2.427**	**1.679-3.51**	**<0.001**
GWG in the second pregnancy	**1.112**	**1.075-1.151**	**<0.001**	**1.137**	**1.095-1.181**	**<0.001**
IPCB	**1.095**	**1.015-1.180**	**0.018**	1.022	0.936-1.117	0.627
GDM in the second pregnancy	**1.718**	**1.197-2.465**	**0.003**	1.236	0.787-1.943	0.358

GDM, gestational diabetes mellitus; LGA, large for gestational age; IPCB, inter-pregnancy change of body mass index; GWG, gestational weight gain; *adjusted factors: previous GDM, nationality, previous LGA, inter-pregnancy interval, IPCB, maternal age in the second pregnancy, GDM in the second pregnancy, pre-pregnancy BMI in the second pregnancy, male newborn in the second pregnancy, GWG in the second pregnancy. Numbers with statistical significance were marked in bold.

After adjusting for potential confounding factors using logistic multivariate regression, previous GDM, LGA, pre-pregnancy BMI, male newborn, and GWG in the second pregnancy were significantly associated with LGA in the second pregnancy (*P*<0.05) (**Table 1**). Collinearity analysis showed that there was no multicollinearity effect between these factors (**Supplementary Table S5**). However, the significant associations of IPI, maternal age and GDM in the second pregnancy with LGA in the second pregnancy were lost in the multivariate regression analysis (**Table 1**). The three-step analysis showed that maternal age in the second pregnancy was a mediator of the association between IPI and LGA (**Supplementary Table S6**, **Supplementary Figure S1**). Moreover, GDM in the first pregnancy confounded the association between GDM in the second pregnancy and LGA (**Supplementary Table S7**, **Supplementary Figure S2**).

Previous GDM, LGA, pre-pregnancy BMI, male newborn, and GWG in the second pregnancy were also significantly associated with macrosomia in the second pregnancy in logistic multivariate regression (*P*<0.05) (**Table 2**). However, the significant associations of IPCB and GDM in the second pregnancy with macrosomia in the second pregnancy were lost in the multivariate regression analysis (**Table 2**). The three-step analysis showed that pre-pregnancy BMI in the second pregnancy was a mediator of the association between IPCB and macrosomia (**Supplementary Table S8**, **Supplementary Figure S3**). Moreover, GDM in the first pregnancy confounded the association between GDM in the second pregnancy and macrosomia (**Supplementary Table S9**, **Supplementary Figure S4**).

### 3.4 The association between previous GDM and the occurrence of LGA and macrosomia varied in different populations

In a stratified logistic multivariate analysis, previous GDM was independently associated with an increased risk of LGA in the second pregnancy among women without prior LGA, with GDM, appropriate GWG, non-advanced pregnancy, and male newborns (*P* < 0.05) (**Table 3**). The adjusted OR values for these subjects (aOR: 1.738, 1.789, 1.926, 1.799, and 1.626) were all higher than that for the overall population (aOR: 1.511). Similarly, previous GDM was independently linked to a heightened risk of macrosomia in the same cohort (*P* < 0.05) (**Table 3**). The adjusted ORs for these subjects (aOR: 2.299, 2.769, 3.198, 2.067, and 2.438) also exceeded those of the overall population (aOR: 1.854). However, among women with previous LGA, EGWG, advanced pregnancy, and female newborns in the second pregnancy, no significant association was found between previous GDM and LGA or macrosomia (**Table 3**). In women without GDM in the second pregnancy, who had significantly higher GWG compared to those GDM women (**Supplementary Table S2**), previous GDM was not significantly associated with the risk of LGA or macrosomia (*P* > 0.05) (**Table 3**). Moreover, no significant interaction between stratification factors and GDM in the first pregnancy was found in the interaction analysis (*P* > 0.05) (**Table 3**).

**Table 3:** Stratified multivariate logistic analysis of previous GDM for LGA and macrosomia in the second pregnancy.

Subgroups for analysis	Effect of previous GDM on LGA in the second pregnancy			Effect of previous GDM on macrosomia in the second pregnancy		
	aOR*	95% CI*	*P* for interaction	aOR*	95% CI*	*P* for interaction
without LGA in previous pregnancy (n=2818)	**1.738**	**1.179-2.562**	0.158	**2.299**	**1.235-4.280**	0.327
with LGA in previous pregnancy (n=313)	0.978	0.457-2.091		1.376	0.574-3.301	
without GDM in the second pregnancy (n=2630)	1.375	0.849-2.225	0.602	1.199	0.549-2.617	0.115
with GDM in the second pregnancy (n=501)	**1.789**	**1.055-3.034**		**2.769**	**1.298-5.907**	
insufficient GWG in the second pregnancy (n=316)	1.052	0.323-3.421	0.195	1.260	0.092-17.258	0.204
appropriate GWG in the second pregnancy (n=1377)	**1.926**	**1.077-3.444**		**3.198**	**1.199-8.525**	
excessive GWG in the second pregnancy (n=1438)	1.448	0.894-2.345		1.626	0.875-3.018	
maternal age less than 35 years in the second pregnancy (n=2214)	**1.799**	**1.169-2.769**	0.081	**2.067**	**1.118-3.823**	0.799
maternal age ≥ 35 years in the second pregnancy (n=917)	1.153	0.629-2.114		1.509	0.600-3.793	
male newborn in the second pregnancy (n=1689)	**1.626**	**1.026-2.578**	0.690	**2.438**	**1.347-4.413**	0.738
female newborn in the second pregnancy (n=1442)	1.409	0.818-2.425		1.122	0.402-3.134	

GDM, gestational diabetes mellitus; LGA, large for gestational age; GWG, gestational weight gain; aOR, adjusted odds ratio; *adjusted factors: previous GDM, nationality, previous LGA, IPI, IPCB, maternal age in the second pregnancy, GDM in the second pregnancy, pre-pregnancy BMI in the second pregnancy, male newborn in the second pregnancy, GWG in the second pregnancy. Numbers with statistical significance were marked in bold.

### 3.5 The impact of GWG on LGA and macrosomia is influenced by prior GDM

**Figure 3:**
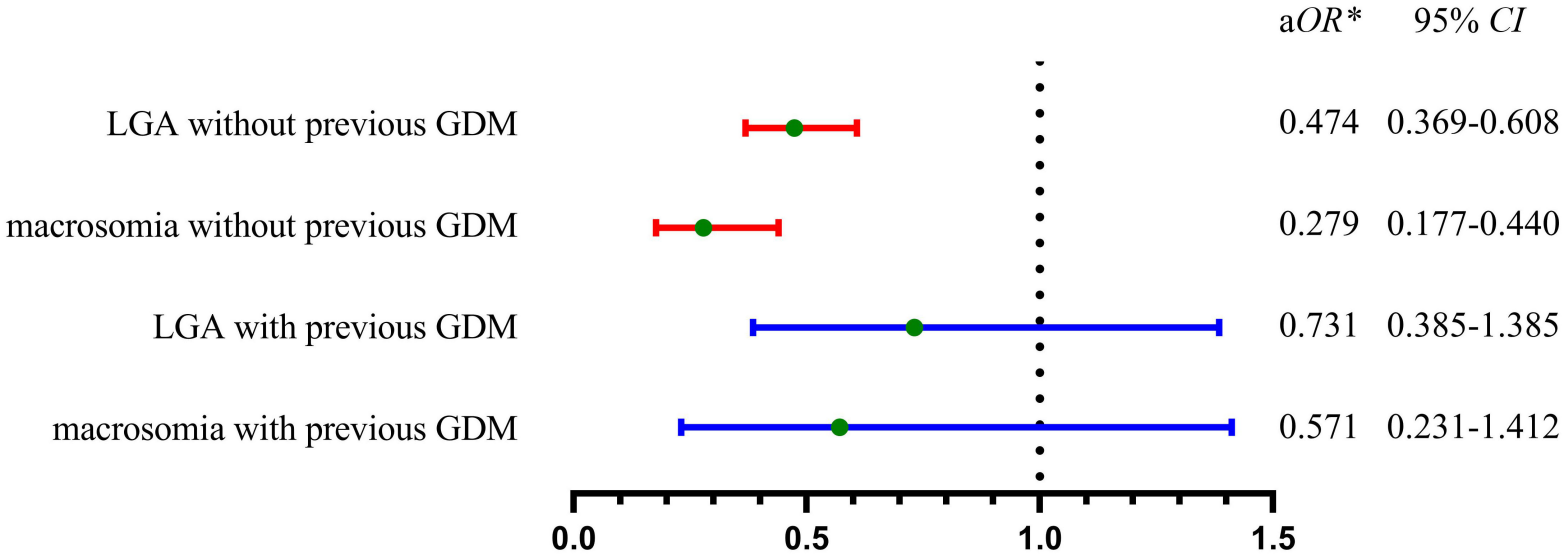
Adjusted odds ratios of AGWG versus EGWG for the risk of LGA and macrosomia in the second pregnancy. In women without previous GDM, AGWG owned significantly lower risk of LGA or macrosomia when compared with EGWG (red line). In women with previous GDM, there was no significant difference of the risk of LGA and macrosomia between AGWG and EGWG (blue line). GDM, gestational diabetes mellitus; LGA, large for gestational age; AGWG, appropriate gestational weight gain; EGWG, excessive gestational weight gain; aOR, adjusted odds ratio; ^*^adjusted by nationality, previous LGA, IPI, IPCB, maternal age in the second pregnancy, GDM in the second pregnancy, pre-pregnancy BMI in the second pregnancy, male newborn in the second pregnancy, GWG in the second pregnancy.

In women without prior GDM, appropriate gestational weight gain (AGWG) was linked to lower risks of LGA and macrosomia in the second pregnancy when compared to excessive gestational weight gain (EGWG) in logistic multivariate analysis (**Figure 3**). Further stratified analyses indicated that the risk of LGA and macrosomia was significantly reduced in AGWG compared with EGWG in the normal weight group before the second pregnancy, while this difference was not significant in the underweight, overweight, or obese groups (**Table 4**).

**Table 4:** Stratified multivariate logistic analysis of previous GDM for LGA and macrosomia in the second pregnancy.

	LGA			Macrosomia		
	n (%)	*χ*²	*P*	n (%)	*χ*²	*P*
GDM^1^+UW+AGWG^2^ (n=20)	2(10.00)	–	0.437^*^	1(5.00)	–	–
GDM^1^+UW+EGWG^2^ (n=4)	1(25.00)			0(0.00)		
GDM^1^+NW+AGWG^2^ (n=92)	13(14.13)	2.007	0.157	5(5.43)	1.938	0.164
GDM^1^+NW+EGWG^2^ (n=90)	20(22.22)			10(11.11)		
GDM^1^+OB+AGWG^2^ (n=20)	10(50.00)	0.033	0.855	4(20.00)	0.682	0.409
GDM^1^+OB+EGWG^2^ (n=40)	21(52.50)			12(30.00)		
non-GDM^1^+UW+AGWG^2^ (n=194)	13(6.70)	3.150	0.076	1(0.52)	–	0.059^*^
non-GDM^1^+UW+EGWG^2^ (n=110)	14(12.73)			4(3.64)		
non-GDM^1^+NW+AGWG^2^ (n=936)	98(10.47)	**30.924**	**<0.001**	19(2.03)	**37.569**	**<0.001**
non-GDM^1^+NW+EGWG^2^ (n=913)	180(19.72)			76(8.32)		
non-GDM^1^+OB+AGWG^2^ (n=115)	22(19.13)	2.746	0.098	9(7.83)	0.441	0.507
non-GDM^1^+OB+EGWG^2^ (n=281)	76(27.05)			28(9.96)		

GDM, gestational diabetes mellitus; LGA, large for gestational age; UW, underweight before the second pregnancy; NW, normal weight before the second pregnancy; OB, overweight or obese before the second pregnancy; AGWG, appropriate gestational weight gain; EGWG, excessive gestational weight gain; ^1^in the first pregnancy; ^2^in the second pregnancy; ^*^ Fisher’s precision probability test. Numbers with statistical significance were marked in bold.

Conversely, for women with a history of GDM, the risk of LGA or macrosomia showed no significant difference whether gestational weight gain was appropriate or excessive (**Figure 3**). Further stratified analysis suggested that no significant difference in the risk of LGA and macrosomia between AGWG and EGWG, regardless of pre-pregnancy BMI classification (underweight, normal, overweight, or obese) (**Table 4**).

## 4 Discussion

This study indicates that previous gestational diabetes mellitus (GDM) is linked to a higher risk of subsequent large for gestational age (LGA) and macrosomia. This relationship was observed in newborns of mothers who did not previously deliver LGA babies, were younger, gained appropriate weight during pregnancy, and had male newborns. Additionally, a history of GDM may hinder a pregnant woman’s ability to mitigate the risk of excessive fetal growth by controlling gestational weight gain (GWG). Over the past decade, the risk of LGA among Chinese women with GDM has remained relatively high, emphasizing the need to identify risk factors and implement effective intervention strategies (2). In the context of China’s new birth policy, the findings of this study underscore the clinical importance of managing GDM in a previous pregnancy to reduce the risk of LGA and macrosomia in subsequent pregnancies.

A previous report from the United States indicated that a history of GDM increases the risk of LGA in subsequent pregnancies (17). However, a recent multicenter study in China did not find this association (18). This study suggested that the lack of association might be due to effective GDM control (18). Considering the recent reports on the birth weight curve (19) and gestational weight gain standards (22) for the Chinese population, there is a growing need to explore the relationship between GDM, LGA, macrosomia, and GWG in this demographic. The impact of gestational diabetes mellitus (GDM) in a prior pregnancy on large-for-gestational-age (LGA) infants in a subsequent pregnancy may be associated with post-pregnancy insulin resistance. Compared to women without a history of GDM, those with such a history exhibit lower insulin sensitivity and impaired β-cell function, leading to subclinical hyperglycemia in their second pregnancy (23). Insulin resistance during the second trimester is linked to an increased risk of LGA, independent of maternal obesity or blood glucose levels (24). Lin et al. (25) proposed that GDM, combined with insulin resistance, heightens the risk of LGA. Furthermore, increased insulin resistance during pregnancy has been correlated with excessive weight gain, macrosomia, and LGA in Chinese women with GDM (26).

Univariate analysis initially indicated an association between IPI, maternal age, GDM in the second pregnancy with LGA in the second pregnancy. However, these relationships were not supported by multivariate analysis. Collinearity analysis confirmed the absence of multicollinearity among these variables. Notably, IPI showed a strong positive correlation with maternal age in the second pregnancy, as revealed by the three-step method. When considering maternal age as a mediator, IPI was not independently linked to LGA in the second pregnancy. Similarly, GDM in the second pregnancy, initially significant in univariate analysis, lost its association with LGA and macrosomia in multivariate analysis, likely due to the confounding effect of GDM in the first pregnancy, which significantly influenced GDM, LGA, and macrosomia in the second pregnancy. It is reported that the effect of pre-pregnancy overweight/obesity on the macrosomia and LGA was partly mediated by GDM (3). These findings underscore the necessity of accounting for interactions among risk factors when examining their influence on LGA in subsequent pregnancies.

In women without a history of LGA delivery, previous GDM is linked to a heightened risk of LGA and macrosomia in subsequent pregnancy. Compared to the general population, this risk is particularly higher in these women (aOR 1.738 vs. 1.511). Conversely, no such correlation is found in women with a history of LGA. This could be attributed to the fact that a history of LGA is a significant risk factor for LGA in future pregnancies (27), where the OR values for LGA and macrosomia in subsequent pregnancy are 6.318 and 5.616, respectively. The influence of GDM might be diminished by the prior LGA, rendering it non-significant. This indicates that GDM’s impact may fluctuate based on the presence or absence of a history of LGA. Women without a history of LGA delivery often represent the majority and are generally perceived to have a lower risk of LGA, yet GDM can still pose significant adverse effects.

A history of GDM significantly increased the risk of LGA and macrosomia in younger women (<35 years), while this association was not observed in advanced pregnancies. According to the multivariate analysis (**Tables 1**, **2**), the age of the second pregnancy was not an independent risk factor for LGA or macrosomia. However, studies have reported that advanced maternal age (1) or maternal age ≥30 years (28) are high risk factors for LGA and macrosomia. Another research indicates that birth weight and macrosomia increase with maternal age, with age 34 being the turning point, and the risk of low birth weight rises after age 36 (29). Animal studies suggest that placental dysfunction may cause an increased risk of fetal growth restriction in older pregnancies (30). Therefore, the effect of GDM history on excessive fetal growth may be weakened in older pregnant women. An early onset of diabetes significantly increases the risk of developing chronic complications and long-term adverse outcomes (31).

Prior GDM makes male fetuses more prone to LGA or macrosomia, unaffected by factors related to female fetuses. Since the sex of the fetus occurs randomly, it is not correlated with either GWG or LGA history. The heightened susceptibility of male fetuses to GDM-associated overgrowth compared to female fetuses may be attributed to sex differences in insulin-like growth factors (32). This is supported by the higher average birth weight of male fetuses compared to female fetuses and their greater propensity for LGA or macrosomia (33). Additionally, sex-specific extracellular miRNA have been linked to fetal growth and development (34). In female fetuses, levels of leptin (35) and the β-cell function index (HOMO-β) (36) in cord blood are higher than in male fetuses, warranting further investigation into their potential connection to LGA risk.

For women with GDM in their second pregnancy, the risk of LGA and macrosomia was significantly associated with a prior history of GDM. However, this association was not significant in women without GDM in their second pregnancy. Recurrent GDM is linked to obesity and insulin resistance (37), which explains the elevated risk of LGA and macrosomia. In women whose second pregnancy was free of GDM, metabolic disorders may have been corrected, rendering the history of GDM insignificant. Surprisingly, the overall multivariate analysis did not show a significant association between GDM in the second pregnancy and LGA or macrosomia (*P* > 0.05). We believe this outcome may be influenced by reverse causality, as women with GDM had significantly lower GWG compared to those without GDM in subsequent pregnancies (11.92 ± 4.19 kg vs 13.50 ± 4.23 kg) (**Supplementary Table S2**). A reduced GWG might protect pregnant women with GDM during their second pregnancy from LGA and macrosomia (38).

The results from stratified analyses suggest that the link between a history of GDM and LGA in the second pregnancy may be confined to specific subgroups. However, this association could also be influenced by the smaller sample sizes within these subgroups, as no significant interaction was found between stratification factors and GDM (P > 0.05). Another study from China also suggests that there was no significant interaction between GDM subtypes and pre-BMI for LGA (39). Expanding the sample size in future follow-up studies would help clarify the current study’s findings. Additionally, the wider 95% confidence intervals observed in these analyses could also be a result of reduced sample sizes after stratification. The variability in the study population and insufficient adjustment for confounding factors might further explain these wide confidence intervals, potentially leading to lower statistical power that obscures significant associations. Consequently, future research should consider multi-center studies with larger samples, incorporating factors such as diet, exercise, and lipid levels, to provide a more comprehensive understanding of the risk factors involved.

Gestational weight gain (GWG) is a significant risk factor for LGA and macrosomia across all BMI categories, especially in overweight and obese women (40). Appropriate gestational weight gain is known to reduce the risk of LGA in women with GDM and obesity (41). Conversely, excessive gestational weight gain (EGWG) increased the risk of LGA (42, 43). In our stratified analyses, a history of GDM was significantly associated with the risk of LGA and macrosomia in the appropriate gestational weight gain (AGWG) group, but not in the EGWG group. The negative outcomes in women with EGWG during their second pregnancy might stem from EGWG obscuring the influence of a previous GDM history on the incidence of LGA and macrosomia.

To reduce the risk of adverse pregnancy outcomes such as LGA and macrosomia, diet (44) and exercise (45) therapy are recommended in clinical practice for controlling gestational weight. However, our study indicates that a history of GDM may influence the effectiveness of weight management. In pregnant women with prior GDM, regardless of their BMI classification before the second pregnancy, the risk of LGA or macrosomia remains significant even if GWG is within the appropriate range. Conversely, in the absence of a GDM history and with a pre-pregnancy BMI within the normal range, maintaining GWG within the recommended limits can significantly reduce the risk of LGA and macrosomia. In overweight or underweight pregnant women with AGWG, the incidence of LGA decreased significantly (from 27.05% to 19.13% and from 12.73% to 6.70%, respectively). However, this reduction is not statistically significant due to the small sample size. This finding suggests that managing GWG to mitigate the risk of excessive fetal growth may be challenging in women with a history of GDM, while it may be more straightforward for those without GDM. A history of GDM is not only linked to an increased risk of LGA and complications in subsequent pregnancies but also affects the efficacy of weight management in mitigating these risks.

Preventing macrosomia involves the early detection of excessive fetal growth and its risk factors. Research suggests that fetal overgrowth related to GDM can be identified as early as 20 weeks of gestation (46). Additionally, blood glucose levels measured between 10 and 14 weeks show a positive correlation with estimated fetal weight from 23 weeks onward, becoming significant by 27 weeks (46). Measurements of fetal abdominal circumference and estimated fetal weight (EFW) at 19-21 weeks’ gestation are considered indicative of GDM in women with specific risk factors, such as a history of gestational diabetes, a pre-pregnancy BMI of 30 kg/m² or higher, or fasting plasma glucose levels between 5.6 and 6.9 mmol/L at the initial prenatal visit (47). Even before a formal GDM diagnosis, the fetus may exhibit accelerated growth directly linked to maternal hyperglycemia (48). Italian guidelines advise GDM screening for these high-risk women between 16 and 18 weeks of gestation to enable timely intervention and risk control for macrosomia (49). Compared to high-risk pregnant women screened for GDM at 24-28 weeks, those screened earlier at 16-18 weeks show smaller fetal abdominal circumferences and estimated weights (50). Furthermore, numerous maternal biological indicators have been proposed as predictors of macrosomia; however, their efficacy in early prediction requires further investigation (51). Certain differential species of maternal gut microbiota in early pregnancy may serve as potential predictors for preventing macrosomia (52). Therefore, for women with a history of GDM, enhanced monitoring of fetal or maternal markers early in the second trimester and earlier GDM screening can aid in identifying fetal overgrowth promptly, allowing for proactive strategies to minimize the incidence of macrosomia and LGA.

There are some limitations in this study. First, this single-center retrospective study spanned over 20 years, and some early cases were excluded due to a lack of GWG or pre-pregnancy BMI data, potentially introducing selection bias. Second, information on diet, exercise, and lipid profiles of the cases was not collected, and the influence of these confounding factors cannot be ruled out. Nevertheless, over 40% of cases showed a stable weight range (± 1kg/m²) between pregnancies, and less than 10% had an IPCB of more than 3 units, suggesting minimal changes in body weight and its related factors. Third, in the stratified analysis, some subgroups had insufficient sample sizes, affecting statistical power and potentially concealing differences. Increasing the sample size is necessary for further exploration. Fourth, the impact of a history of GDM on the association between GWG and the risk of LGA and macrosomia is based solely on retrospective observational data and requires confirmation through prospective intervention studies.

In conclusion, GDM in previous pregnancy is an independent risk factor for LGA and macrosomia in subsequent pregnancies, as indicated by this study. However, this relationship is influenced by factors such as GWG, prior LGA history, fetal sex, and maternal age. Managing weight alone may not sufficiently lower the risk of LGA or macrosomia in women with a history of GDM. Following the new birth policy in China, the proportion of multipara and advanced pregnancy has increased, leading to a higher incidence of GDM, LGA, and macrosomia. The study’s findings indicate a critical time window for controlling the risks of LGA and macrosomia. Mitigating the risk of GDM in a previous pregnancy can reduce the likelihood of LGA and macrosomia in subsequent pregnancies. Given the limitations of this single-center, retrospective study, a prospective multicenter study is necessary to verify these results further.

## Data Availability

The raw data supporting the conclusions of this article will be made available by the authors, without undue reservation.
