# Supplementary material for: Gestational diabetes mellitus in previous pregnancy associated with the risk of large for gestational age and macrosomia in the second pregnancy
Source: Front Endocrinol (Lausanne). 2025 Feb 3;16:1474694. doi: 10.3389/fendo.2025.1474694 (PMC11830583; doi:10.3389/fendo.2025.1474694)
Supplement: Supplementary file 5 [file Table1.docx]

Table S1 Comparison of the complications and comorbidities between the groups divided by the GDM in the first pregnancy

| Risk factors |  | GDM in the first pregnancy (n=322) | non-GDM in the first pregnancy (n=2809) | χ² | *P* |
| --- | --- | --- | --- | --- | --- |
| GDM in the second pregnancy | | 180(55.9) | 321(11.43) | 425.100 | ＜0.001 |
| preterm birth in the second pregnancy | | 17(5.28) | 104(3.7) | 1.934 | 0.164 |
| PROM in the second pregnancy | | 45(13.98) | 425(15.13) | 0.302 | 0.583 |
| placental abruption | | 3(0.93) | 10(0.36) | - | 0.142 |
| thyroid disease in the second pregnancy | | 31(9.63) | 248(8.83) | 0.227 | 0.634 |
| uterine inertia | | 16(4.97) | 110(3.92) | 0.829 | 0.362 |
| fetal distress | | 3(0.93) | 25(0.89) | - | 0.762 |
| meconium-stained amniotic fluid | | 2(0.62) | 49(1.74) | 2.275 | 0.131 |
| neonatal asphyxia | | 1(0.31) | 13(0.46) | - | 1.000 |

GDM: gestational diabetes mellitus; PROM: premature rupture of membrane.
