# Supplementary material for: Gestational diabetes mellitus in previous pregnancy associated with the risk of large for gestational age and macrosomia in the second pregnancy
Source: Front Endocrinol (Lausanne). 2025 Feb 3;16:1474694. doi: 10.3389/fendo.2025.1474694 (PMC11830583; doi:10.3389/fendo.2025.1474694)
Supplement: Supplementary file 6 [file Table2.docx]

Table S2 Comparison of the GWG in the second pregnancy between subgroups divided by different factors

|  | GWG in the second pregnancy (kg) | *t* | *P* |
| --- | --- | --- | --- |
| GDM in previous pregnancy (n=322) | 12.08±4.35 | 5.232 | ＜0.001 |
| non-GDM in previous pregnancy (n=2809) | 13.38±4.23 |  |  |
|  |  |  |  |
| LGA in previous pregnancy (n=313) | 13.64±4.66 | 1.576 | 0.116 |
| non-LGA in previous pregnancy (n=2818) | 13.21±4.21 |  |  |
|  |  |  |  |
| GDM in the second pregnancy (n=501) | 11.92±4.19 | 7.681 | ＜0.001 |
| non-GDM in the second pregnancy (n=2630) | 13.50±4.23 |  |  |
|  |  |  |  |
| AP in the second pregnancy (n=917) | 13.10±4.23 | 1.267 | 0.205 |
| Non-AP in the second pregnancy (n=2214) | 13.31±4.27 |  |  |
|  |  |  |  |
| Male newborn in the second pregnancy (n=1689) | 13.18±4.25 | 1.018 | 0.309 |
| Female newborn in the second pregnancy (n=1442) | 13.33±4.28 |  |  |
|  |  |  |  |
| underweight in the second pregnancy (n=423) | 13.87±4.31 | 2.002^*^ | 0.045^*^ |
| nomal weight in the second pregnancy (n=2193) | 13.43±4.09 | 6.501^**^ | ＜0.001^**^ |
| overweight or obesity in the second pregnancy (n=515) | 11.97±4.69 | 6.448^***^ | ＜0.001^***^ |

GDM: gestational diabetes mellitus; LGA: large for gestational age; ^*^compared between the underweight and the normal weight; ^**^compared between the normal weight and overweight or obesity; ^***^compared between the underweight and the overweight or obesity.
