# Supplementary material for: Gestational diabetes mellitus in previous pregnancy associated with the risk of large for gestational age and macrosomia in the second pregnancy
Source: Front Endocrinol (Lausanne). 2025 Feb 3;16:1474694. doi: 10.3389/fendo.2025.1474694 (PMC11830583; doi:10.3389/fendo.2025.1474694)
Supplement: Supplementary file 7 [file Table3.docx]

Table S3 Effect of the nationality and IPCB on LGA in the second pregnancy

| risk factors | non-adjusted | | |  | adjusted* | | |
| --- | --- | --- | --- | --- | --- | --- | --- |
|  | *OR* | *95% CI* for *OR* | *P* |  | *OR* | *95% CI* for *OR* | *P* |
| Han nationality | 1.682 | 0.940-3.011 | 0.080 |  | 1.636 | 0.879-3.048 | 0.121 |
| IPCB | 1.050 | 0.998-1.106 | 0.062 |  | 0.992 | 0.932-1.056 | 0.808 |

LGA: large for gestational age; IPCB: inter-pregnancy change of body mass index; *adjusted factors: GDM in previous pregnancy, nationality, LGA in previous pregnancy, inter-pregnancy interval, IPCB, maternal age in the second pregnancy, GDM in the second pregnancy, pre-pregnancy BMI in the second pregnancy, male newborn in the second pregnancy, gestational weight gain in the second pregnancy.
