# Supplementary material for: Gestational diabetes mellitus in previous pregnancy associated with the risk of large for gestational age and macrosomia in the second pregnancy
Source: Front Endocrinol (Lausanne). 2025 Feb 3;16:1474694. doi: 10.3389/fendo.2025.1474694 (PMC11830583; doi:10.3389/fendo.2025.1474694)
Supplement: Supplementary file 8 [file Table4.docx]

Table S4 Effect of the nationality, IPI and maternal age in the second pregnancy on macrosomia in the second pregnancy

| risk factors | non-adjusted | | |  | Adjusted* | | |
| --- | --- | --- | --- | --- | --- | --- | --- |
|  | *OR* | *95% CI* for *OR* | *P* |  | *OR* | *95% CI* for *OR* | *P* |
| Han nationality | 2.468 | 0.777-7.839 | 0.125 |  | 2.384 | 0.709-8.019 | 0.160 |
| IPI | 1.004 | 0.999-1.009 | 0.106 |  | 1.005 | 0.998-1.012 | 0.180 |
| Maternal age in the second pregnancy | 1.021 | 0.980-1.064 | 0.324 |  | 0.975 | 0.924-1.029 | 0.358 |

IPI: inter-pregnancy interval; *adjusted factors: GDM in previous pregnancy, nationality, LGA in previous pregnancy, IPI, inter-pregnancy change of body mass index, maternal age in the second pregnancy, GDM in the second pregnancy, pre-pregnancy BMI in the second pregnancy, male newborn in the second pregnancy, gestational weight gain in the second pregnancy.
