# Supplementary material for: Gestational diabetes mellitus in previous pregnancy associated with the risk of large for gestational age and macrosomia in the second pregnancy
Source: Front Endocrinol (Lausanne). 2025 Feb 3;16:1474694. doi: 10.3389/fendo.2025.1474694 (PMC11830583; doi:10.3389/fendo.2025.1474694)
Supplement: Supplementary file 9 [file Table5.docx]

Table S5 Collinearity analysis of the independent variables

| variables | tolerance | VIF |
| --- | --- | --- |
| GDM in previous pregnancy | 0.840 | 1.190 |
| Han nationality | 0.993 | 1.007 |
| LGA in previous pregnancy | 0.959 | 1.043 |
| IPI | 0.700 | 1.429 |
| IPCB | 0.717 | 1.394 |
| Maternal age in the second pregnancy | 0.703 | 1.423 |
| GDM in the second pregnancy | 0.827 | 1.209 |
| Pre-pregnancy BMI in the second pregnancy | 0.710 | 1.407 |
| Male newborn in the second pregnancy | 0.996 | 1.004 |
| GWG in the second pregnancy | 0.934 | 1.071 |

GDM: gestational diabetes mellitus; LGA: large for gestational age; IPI: inter-pregnancy interval; IPCB: inter-pregnancy change of body mass index; GWG: gestational weight gain; VIF: variance inflation factor.
