# Supplementary material for: Gestational diabetes mellitus in previous pregnancy associated with the risk of large for gestational age and macrosomia in the second pregnancy
Source: Front Endocrinol (Lausanne). 2025 Feb 3;16:1474694. doi: 10.3389/fendo.2025.1474694 (PMC11830583; doi:10.3389/fendo.2025.1474694)
Supplement: Supplementary file 10 [file Table6.docx]

Table S6 The effect of maternal age in the second pregnancy as a mediator on the correlation between IPI and LGA in subsequent pregnancy

| Steps | Factors in analysis | Statistics method | OR or *t* | 95% CI or *P* |
| --- | --- | --- | --- | --- |
| Step 1 | IPI on LGA^2^ | Univariate analysis | **1.004** | **1.001-1.007** |
|  |  |  |  |  |
| Step 2 | IPI on Maternal age^2^ | Linear regression | ***t*=34.688** | ***P*＜0.001** |
|  | Maternal age^2^ on LGA^2^ | Univariate analysis | **1.042** | **1.016-1.070** |
|  |  |  |  |  |
| Step 3 | IPI on the LGA^2^ | multivariate logistic regression^*^ | 1.002 | 0.998-1.006 |
|  | Maternal age^2^ on LGA^2^ | multivariate logistic regression^*^ | **1.034** | **1.003-1.066** |

LGA: large for gestational age; IPI: inter-pregnancy interval;^*^ adjusted by IPI and the maternal age in the second pregnancy; ^1^ in the first pregnancy; ^2^ in the second pregnancy.
