# Supplementary material for: Gestational diabetes mellitus in previous pregnancy associated with the risk of large for gestational age and macrosomia in the second pregnancy
Source: Front Endocrinol (Lausanne). 2025 Feb 3;16:1474694. doi: 10.3389/fendo.2025.1474694 (PMC11830583; doi:10.3389/fendo.2025.1474694)
Supplement: Supplementary file 11 [file Table7.docx]

Table S7 The effect of GDM in the first pregnancy as a confounding factor on the correlation between GDM in the second pregnancy and LGA in subsequent pregnancy

| Steps | Factors in analysis | Statistics method | OR | 95% CI |
| --- | --- | --- | --- | --- |
| Step 1 | GDM^1^ on LGA^2^ | Univariate analysis | **1.374** | **1.077-1.753** |
|  |  |  |  |  |
| Step 2 | GDM^1^ on GDM^2^ | Univariate analysis | ***9.825*** | **7.661-12.600** |
|  | GDM^2^ on LGA^2^ | Univariate analysis | **1.618** | **1.222-2.141** |
|  |  |  |  |  |
| Step 3 | GDM^1^ on LAG^2^ | multivariate logistic regression^*^ | **1.485** | **1.094-2.017** |
|  | GDM^2^ on LGA^2^ | multivariate logistic regression^*^ | 1.208 | 0.926-1.576 |

GDM: gestational diabetes mellitus; LGA: large for gestational age;^*^ adjusted by GDM in the first pregnancy and GDM in the second pregnancy; ^1^ in the first pregnancy; ^2^ in the second pregnancy.
