# Supplementary material for: Gestational diabetes mellitus in previous pregnancy associated with the risk of large for gestational age and macrosomia in the second pregnancy
Source: Front Endocrinol (Lausanne). 2025 Feb 3;16:1474694. doi: 10.3389/fendo.2025.1474694 (PMC11830583; doi:10.3389/fendo.2025.1474694)
Supplement: Supplementary file 12 [file Table8.docx]

Table S8 The effect of pre-pregnancy BMI in the second pregnancy as a mediator on the correlation between IPCB and macrosomia in subsequent pregnancy

| Steps | Factors in analysis | Statistics method | OR or *t* | 95% CI or *P* |
| --- | --- | --- | --- | --- |
| Step 1 | IPCB on macrosomia^2^ | Univariate analysis | **1.095** | **1.015-1.180** |
|  |  |  |  |  |
| Step 2 | IPCB on pre-pregnancy BMI^2^ | Linear regression | ***t*=29.717** | ***P*＜0.001** |
|  | pre-pregnancy BMI^2^ on macrosomia^2^ | Univariate analysis | **1.186** | **1.134-1.241** |
|  |  |  |  |  |
| Step 3 | IPCB on the macrosomia^2^ | multivariate logistic regression^*^ | 0.936 | 0.864-1.015 |
|  | pre-pregnancy BMI^2^ on macrosomia^2^ | multivariate logistic regression^*^ | **1.213** | **1.152-1.278** |

LGA: large for gestational age; IPCB: inter-pregnancy change of body mass index;^*^ adjusted by IPCB and the pre-pregnancy BMI in the second pregnancy; ^1^ in the first pregnancy; ^2^ in the second pregnancy.
